# Associations between Left Ventricular Cavity Size and Cardiac Function and Overload Determined by Natriuretic Peptide Levels and a Covariance Structure Analysis

**DOI:** 10.1038/s41598-017-02247-5

**Published:** 2017-05-17

**Authors:** Jun Yoshida, Makoto Kawai, Kosuke Minai, Kazuo Ogawa, Takayuki Ogawa, Michihiro Yoshimura

**Affiliations:** Division of Cardiology, Department of Internal Medicine, The Jikei University School of Medicine, 3-25-8 Nishi-shimbashi, Minato-ku, Tokyo 105-8461 Japan

## Abstract

The effects of left ventricular (LV) cavity size on cardiac function and overload have not yet been fully elucidated. We performed a covariance structure analysis and drew theoretical path models to clarify the effects of hemodynamic parameters on the stroke volume index (SVI) as a marker of cardiac function and on the plasma B-type natriuretic peptide (BNP) level as a marker of cardiac overload. We simultaneously measured various hemodynamic parameters and the BNP levels during cardiac catheterization in 1,715 inpatients of our institution. The current path models tested the validity of the Frank-Starling law in patients with heart failure using the SVI, the LV end-systolic volume index (LVESVI) and the LV end-diastolic volume index (LVEDVI). Using the BNP levels, the path models clearly demonstrated that LVESVI substantially augmented cardiac overload, whereas LVEDVI palliated this parameter. These volume indices exerted opposite effects on cardiac function and overload. These results advance the understanding of the relationships between LV cavity size and both cardiac function and overload and indicate the increasing importance of LV diastolic volume in heart failure and the utility of LVESVI as an important marker of cardiac remodeling for further relevant studies.

## Introduction

The relationships between left ventricular (LV) cavity size and both cardiac function and overload in patients with heart failure are important research questions. The Frank-Starling law explains the significance of LV diastolic cavity size in maintaining cardiac output. Increases in LV end-diastolic volume are known to be compensatory and favorable phenomena for the preservation of cardiac output in some cases of cardiac dysfunction.

However, an association between cardiac function and cardiac overload has not been clearly identified in patients with heart failure, and this remains a pressing issue in cardiac research. Cardiac stress might increase in relation to an increase in LV cavity size, but it remains unclear whether myocardial stress increases within or outside the physiological range when the left ventricle enlarges according to the Frank-Starling law^1^. If the wall stress exceeds the physiological range, further stress due to neurohumoral activation and oxidative stress can occur in the myocardium^2–4^. Furthermore, it is widely believed that cardiac remodeling occurs both histogenetically and morphologically in most patients with cardiovascular diseases^5, 6^, although the extent of cardiac remodeling that occurs in individual patients remains unclear.

The concept of systolic volume is relatively easy to understand in terms of general morphology because the end-systolic left ventricle enlarges as a direct result of systolic dysfunctions caused by some underlying cardiac disorders. Furthermore, the end-systolic left ventricle volume increased because of cardiac remodeling. In both cases, increases in the end-systolic left ventricle volume *per se* should always react adversely to maintain cardiac output. In contrast, the association between cardiac function and cardiac overload during the diastolic phase is difficult to understand and still requires further research. It is unclear whether increases in LV diastolic volume are harmful in terms of myocardial stress in any practical sense. A more complete evaluation and understanding of LV function in patients with heart failure requires more comprehensive assessments, including assessments of LV geometry, LV cavity size, and left atrial size^7^.

As described above, the significance of diastolic volume remains a controversial subject of discussion, and thus we require a more precise examination of the associations between cavity size, especially the end-diastolic LV volume, and both cardiac performance and overload. This apparently straightforward question is actually very difficult to answer in relation to heart failure. One reason for this difficulty is that there is no suitable and sensitive index that represents cardiac overload.

B-type natriuretic peptide (BNP) is a cardiac hormone that is secreted mainly from the ventricle in patients with heart failure^8–10^. BNP is primarily secreted in response to mechanical stretching of the myocardium, although neurohumoral factors can also induce its secretion^11–13^. Plasma BNP levels are believed to be a sensitive and reliable biomarker of the degree of heart failure in clinical practice^14–17^. It is widely accepted that the plasma BNP levels increase in proportion to the severity of not only systolic dysfunction but also diastolic dysfunction^18^. We believe that BNP can be applied as a sensitive biomarker of cardiac overload and thus examined this issue in the present study.

Nonetheless, it would be statistically difficult to determine the individual effect of each parameter among the many factors in actual patients with heart failure because, in general, most hemodynamic parameters are significant confounding factors. Additionally, the plasma BNP levels correlate with the relevant hemodynamic parameters to various degrees^10^. It would be ideal to answer these questions simultaneously by using one equation model. However, the relevant statistics can be rigid and challenging. Such an analysis cannot be performed with commonly used statistical procedures such as multivariate analysis. Herein, we propose that covariance structure analysis is appropriate for addressing this question. In many areas, covariance structure analysis plays an important role in understanding how the relationships between the observed variables might be generated by an interaction effect and/or hypothesized latent variables. Recently, we successfully analyzed complex paths with covariance structure analysis^19^.

In the present study, we examined the effects of individual hemodynamic parameters, particularly the LV end-diastolic volume, on cardiac function and overload in patients with heart failure using a novel combination of plasma BNP levels and covariance structure analysis.

## Results

### Patient characteristics

Table 1 presents the patient characteristics of all patients in Group I (n = 1,715). There were 1,363 patients with ischemic heart disease (IHD; Group II), including 1,080 patients with chronic stable IHD (Group III) and 352 patients without IHD (Group IV).

**Table 1:** The characteristics of all patients.

Characteristics	Overall (n = 1715)	IHD (n = 1363)	Non-IHD (n = 352)
	Number (%) or Mean ± SD [Median; interquartile range]		
Gender; Male	1397 (81.5)	1161 (85.2)	236 (67.0)
Age (years old)	64.6 ± 11.5	65.1 ± 10.9	62.7 ± 13.4
BMI (kg/m^2^)	24.3 ± 4.1	24.6 ± 4.0	23.3 ± 4.4
Current smoker	386 (22.5)	313 (23.0)	73 (20.7)
Family history of IHD	445 (26.1)	372 (27.3)	73 (20.7)
Hb (g/dL)	13.3 ± 2.0	13.3 ± 1.9	13.5 ± 2.3
Creatinine (mg/dL)	1.66 ± 2.54	1.72 ± 2.65	1.42 ± 2.03
eGFR (mL/min/1.73 m^2^)	64.8 ± 26.3	64.9 ± 26.6	64.6 ± 25.1
UA (mg/dL)	6.0 ± 1.5	5.9 ± 1.4	6.3 ± 1.9
FBS (mg/dL)	120.4 ± 39.6	123.1 ± 41.4	110.0 ± 29.0
HbA1c (%)	6.2 ± 1.1	6.3 ± 1.1	5.9 ± 0.9
TG (mg/dL)	124.5 ± 103.7	127.9 ± 110.9	111.3 ± 67.9
HDL-C (mg/dL)	51.9 ± 15.7	51.1 ± 15.2	55.2 ± 16.9
LDL-C (mg/dL)	102.9 ± 31.4	101.4 ± 30.8	108.4 ± 33.1
LDL-C/HDL-C	2.14 ± 0.92	2.14 ± 0.87	2.15 ± 1.08
CRP (mg/dL)	0.58 ± 1.61	0.57 ± 1.66	0.61 ± 1.41
BNP (pg/mL)	[48.0; 17.4–153.8]	[38.7; 15.4–123.3]	[103.7; 34.6–243.0]
**Left ventricular hemodynamic parameters**			
LVEDVI (mL/m^2^)	68.4 ± 23.9	64.8 ± 19.3	82.0 ± 33.2
LVESVI (mL/m^2^)	31.6 ± 19.4	28.9 ± 15.7	42.0 ± 27.4
LVEF (%)	56.1 ± 12.1	57.2 ± 10.8	51.8 ± 15.3
LVEDP (mmHg)	17.1 ± 8.5	16.9 ± 8.7	17.9 ± 7.8
SVI (ml/m²)	39.2 ± 12.2	39.2 ± 12.0	39.2 ± 12.3
**Underlying cardiovascular disease**			
Ischemic heart disease	1363 (79.5)	1363	—
Acute coronary syndrome	113 (6.6)	113 (8.3)	—
Angina pectoris	1216 (70.9)	1216 (89.2)	—
Cardiomyopathy	168 (9.8)	33 (2.4)	135 (38.4)
Valvular disease	191 (11.1)	71 (5.2)	120 (34.1)
Arrhythmia	133 (7.8)	58 (4.3)	75 (21.3)
Atrial fibrillation	112 (6.5)	44 (3.2)	68 (19.3)
Other than AF	21 (1.2)	14 (1.0)	7 (2.0)
Hypertension	1269 (74.0)	1049 (77.0)	220 (62.5)
Type-2 diabetes mellitus	647 (37.7)	566 (41.5)	81 (23.0)
Dyslipidemia	1239 (72.2)	1077 (79.0)	162 (46.0)
Renal dysfunction*	554 (32.3)	428 (31.4)	126 (35.8)
Hemodialysis	179 (10.4)	150 (11.0)	29 (8.2)
**Medication**			
Antiplatelet agent	1158 (67.5)	1057 (77.5)	101 (28.7)
Anticoagulant agent	170 (9.9)	101 (7.4)	69 (19.6)
ACE inhibitors	301 (17.6)	256 (18.8)	45 (12.8)
ARBs	647 (37.7)	545 (40.0)	102 (29.0)
Beta blockers	633 (36.9)	537 (40.0)	96 (27.3)
Calcium channel blockers	923 (53.8)	804 (59.0)	119 (33.8)
Diuretics	320 (18.7)	216 (15.8)	104 (29.5)
Statins	904 (52.7)	824 (60.5)	80 (22.7)
Non- Statin for dyslipidemia	223 (13.0)	199 (14.6)	24 (6.8)
Oral antidiabetic agents	402 (23.4)	355 (26.0)	47 (13.4)
Insulin	177 (10.3)	157 (11.5)	20 (5.7)
Anti-hyperuricemia	280 (16.3)	220 (16.1)	60 (17.0)

Hb, hemoglobin; UA, uric acid; FBS, fasting blood sugar; HbA1c, hemoglobin A1c; TG, triglycerides; HDL-C, high-density lipoprotein; LDL-C, low-density lipoprotein; ACE, angiotensin-converting enzyme; ARBs, angiotensin II type I-receptor blockers; BMI, body mass index; BNP, B-type natriuretic peptide; CRP, C-reactive protein; eGFR, estimated glomerular filtration rate; IHD, ischemic heart disease; LVEDP, left ventricular end-diastolic pressure; LVEDVI, left ventricular end-diastolic volume index; LVEF, left ventricular ejection fraction; LVESVI, left ventricular end-systolic volume index; and SVI, stroke volume index.

*Renal dysfunction = eGFR < 60 mL/min/1.73 m^2^.

### Single regression analyses of the hemodynamic parameters with the log-transformed BNP

We examined the correlations between the LV end-systolic and end-diastolic volume indices (LVESVI and LVEDVI, respectively), the LV end-diastolic pressure (LVEDP), the stroke volume index (SVI), and the log-transformed BNP (Log BNP). Supplemental Table 1 presents the correlations between the variables. Most of the pairs were significant, except for the SVI-LVEDVI and SVI-LVEDP pairs. These findings indicated that the majority of the factors were statistically confounded; thus, we analyzed the relationships between the individual values with covariance structure analysis as follows.

### Concepts and results of the proposed path models A and B

**Figure 1:**
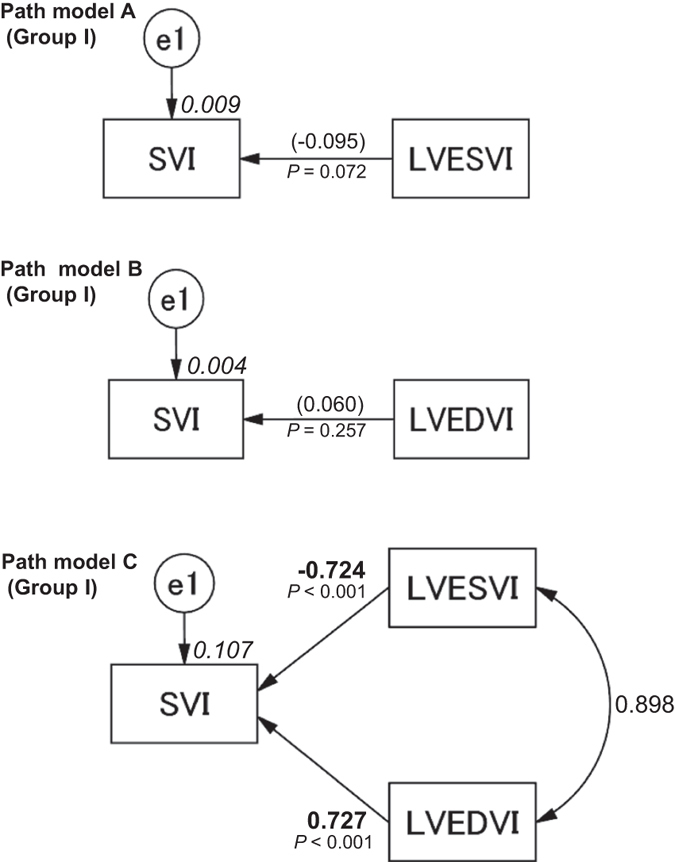
Path models A–C. The paths are displayed with coefficients that indicate the standardized coefficient of the regressing independent variable on the dependent variable of the relevant path (path model A, LVESVI to SVI; path model B, LVEDVI to SVI; path model C, LVESVI and/or LVEDVI to SVI) in Group I. These variables are standardized regression coefficients [direct effect; non-significant coefficients (inside round brackets)], squared multiple correlations [on the upper right side of rectangles (in italics)] and the correlations between each of the variables. Other variables that were included in the covariance structure analysis are presented in Supplemental Tables 2, 3 and 4. LVESVI, left ventricular end-systolic volume index; LVEDVI, left ventricular end-diastolic volume index; SVI, systolic volume index; and e, extraneous variable.

The analysis was initiated using Group I. The first path models, A and B, were proposed as illustrated in Fig. 1 (upper and middle). The paths between the variables were drawn from the independent to the dependent variables, with directional arrows for the regression models. This approach was based on the concept of whether LVESVI or LVEDVI exerted causative effects on SVI. The results of this statistical analysis are presented in Supplemental Tables 2 and 3. The simple path model for the confirmation factor analysis revealed that neither LVESVI (standardized regression coefficients, *β*: −0.095, 95% confidence interval [CI], [−0.168, −0.020], *P* = 0.072) nor LVEDVI influenced SVI (*β*: 0.060, 95% CI [−0.018, 0.136], *P* = 0.257).

### Concept and results of the proposed path model C

The next theoretical path model proposed is illustrated in Fig. 1 (lower). Path model C was drawn in a parallel manner with LVESVI and LVEDVI. The paths were drawn from the independent to the dependent variables with directional arrows for each regression model, i.e., from LVESVI and LVEDVI to SVI. The associations between the factors (LVESVI and LVEDVI) are illustrated with two-way arrows. The results of this statistical analysis are presented in Supplemental Table 4. A theoretical path model based on covariance structure analysis using two dependent variables in the same equation model was proposed. The final model produced the following regression weights after standardizing all of the variables: SVI was predicted by LVESVI (*β*: −0.724, 95% CI [−0.863, −0.569], *P* < 0.001) and LVEDVI (*β*: 0.727, 95% CI [0.569, 0.871], *P* < 0.001). We noted that LVESVI and LVEDVI were inversely and positively associated with SVI, respectively. We believe that this model dovetails with the Frank-Starling law.

### Concept and results of the proposed path models D and E

**Figure 2:**
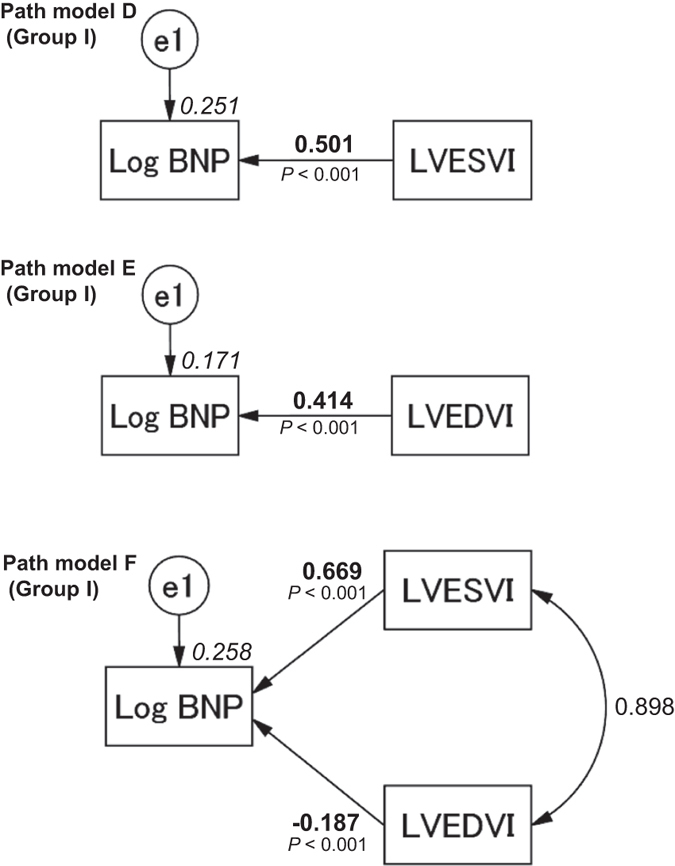
Path models D–F. The paths are displayed with the coefficients that indicate the standardized coefficient of the regressing independent variable on the dependent variable of the relevant path (path model D, LVESVI to Log BNP; path model E, LVEDVI to Log BNP; path model F, LVESVI and/or LVEDVI to Log BNP) in Group I. These variables are the standardized regression coefficients (direct effect), squared multiple correlations [on the upper right side of rectangles (in italics)] and the correlations between each of the variables. Other variables included in the covariance structure analysis are presented in Supplemental Tables 5, 6, and 7. BNP, B-type natriuretic peptide; LVESVI, left ventricular end-systolic volume index; LVEDVI, left ventricular end-diastolic volume index; and e, extraneous variable.

Simple path models D and E were proposed as illustrated in Fig. 2 (upper and middle). The paths were drawn from the independent to the dependent variables with directional arrows that demonstrate each regression model. These models were based on whether LVESVI or LVEDVI causatively induced increases in the plasma BNP levels. The results of these statistical analyses are presented in Supplemental Tables 5 and 6. The simple path model for the confirmation factor analysis revealed that LVESVI (*β*: 0.501, 95% CI [0.465, 0.536], *P* < 0.001) and LVEDVI were causatively related to Log BNP (*β*: 0.414, 95% CI [0.374, 0.453], *P* < 0.001).

### Concept and results of the proposed path model F

Next, the theoretical path model illustrated in Fig. 2 (lower) was proposed. The path model was drawn in a parallel manner with LVESVI and LVEDVI. The paths were drawn from the independent to the dependent variables with directional arrows that were used for each regression model, i.e., the models from LVESVI and LVEDVI to Log BNP. The association between the factors LVESVI and LVEDVI was indicated with two-way arrows. The results of this statistical analysis are presented in Supplemental Table 7. The final model produced the following regression weights after standardizing all of the variables: the Log BNP was predicted by LVESVI (*β*: 0.669, 95% CI [0.580, 0.756], *P* < 0.001) and LVEDVI (*β*: −0.187, 95% CI [−0.278, −0.094], *P* < 0.001). We noted that LVESVI and LVEDVI produced precisely opposite effects on cardiac overload as estimated with the Log BNP.

### Concept and results of the proposed path model G

**Figure 3:**
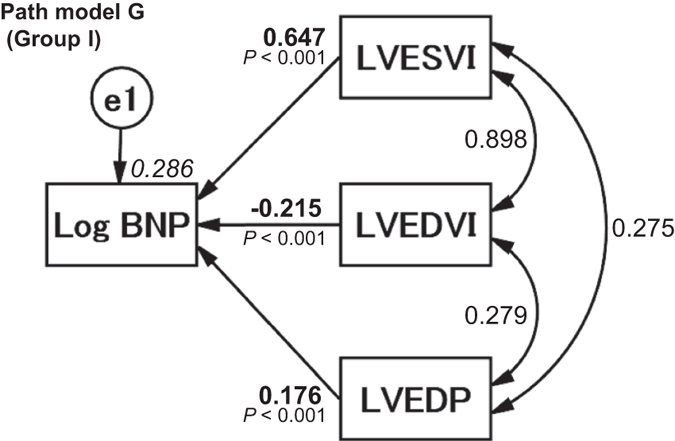
Path model G. The paths are displayed with the coefficients that indicate the standardized coefficient of the regressing independent variable on the dependent variable of the relevant path (path model G, LVESVI and/or LVEDVI and/or LVEDP to Log BNP) in Group I. These variables are the standardized regression coefficients (direct effect), squared multiple correlations [on the upper right side of rectangles (in italics)] and the correlations between each of the variables. Other variables included in the covariance structure analysis are presented in Supplemental Table 8. BNP, B-type natriuretic peptide; LVESVI, left ventricular end-systolic volume index; LVEDVI, left ventricular end-diastolic volume index; LVEDP, left ventricular end-diastolic pressure; and e, extraneous variable.

The theoretical path model illustrated in Fig. 3 was proposed. LVEDP might stimulate increases in plasma BNP levels. Because LVESVI, LVEDVI, and LVEDP were found to confound each other in the simple regression analysis (Supplemental Table 1), path model “G” was drawn with LVESVI, LVEDVI, and LVEDP in a parallel manner. The paths were drawn from the independent to dependent variables with directional arrows for each regression model, i.e., from LVESVI, LVEDVI, and LVEDP to Log BNP. The associations between the pairs of the factors were linked by two-way arrows. The results of this statistical analysis are presented in Supplemental Table 8. The model produced the following regression weights after standardizing all of the variables: the Log BNP was predicted by LVESVI (*β*: 0.647, 95% CI [0.558, 0.733], *P* < 0.001), LVEDVI (*β*: −0.215, 95% CI [−0.306, −0.124], *P* < 0.001) and LVEDP (*β*: 0.176, 95% CI [0.134, 0.217], *P* < 0.001). Importantly, we noted that LVESVI and LVEDVI were positively and inversely associated with Log BNP, respectively. This result was similar to that obtained from path model F (Supplemental Table 7). Additionally, we found that LVEDP was an additional factor that was associated with an increased Log BNP irrespective of LVESVI and LVEDVI.

### Analyses of the other patient groups

**Figure 4:**
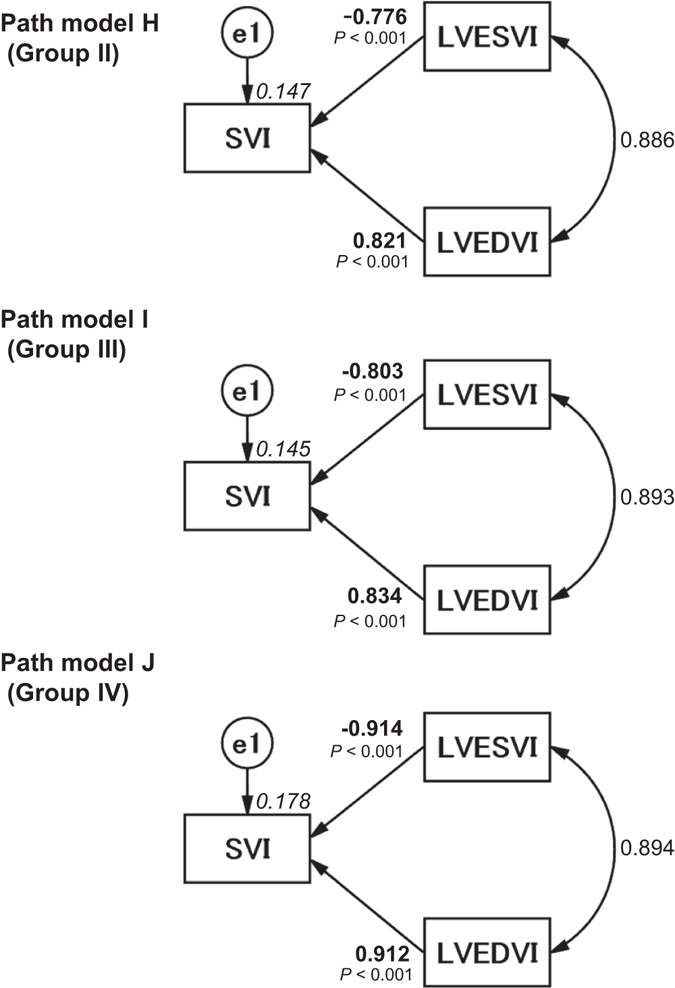
Path models H–J. The paths are displayed with coefficients that indicate the standardized coefficients of the regressing independent variable on the dependent variable of the relevant path in the subtype group (path models H–J, LVESVI and/or LVEDVI to SVI), i.e., Groups II (path model H), III (path model I) and IV (path model J). These variables are the standardized regression coefficients (direct effect), squared multiple correlations [on the upper right side of rectangles (in italics)] and correlations between each of the variables. Other variables included in the covariance structure analysis are shown in Supplemental Tables 9, 10, and 11. LVESVI, left ventricular end-systolic volume index; LVEDVI, left ventricular end-diastolic volume index; SVI, systolic volume index; and e, extraneous variable.

**Figure 5:**
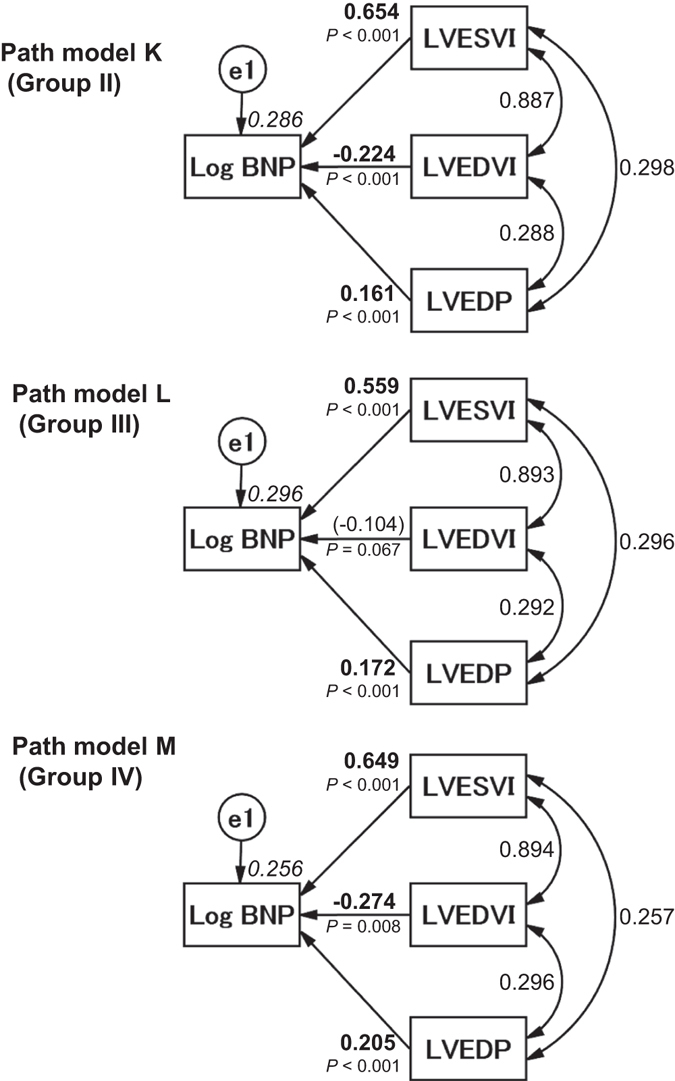
Path models K–M. The paths are displayed with coefficients that indicate the standardized coefficient of the regressing independent variable on the dependent variable of the relevant path in the subtype groups (path models K–M, LVESVI and/or LVEDVI and/or LVEDP to Log BNP) of Groups II (path model K), III (path model L) and IV (path model M). These variables are the standardized regression coefficients [direct effect; not significant coefficients (inside round brackets)], squared multiple correlations [on the upper right side of rectangles (in italics)] and the correlations between each of the variables. Other variables used in the covariance structure analysis are shown in Supplemental Tables 12, 13, and 14. BNP, B-type natriuretic peptide; LVESVI, left ventricular end-systolic volume index; LVEDVI, left ventricular end-diastolic volume index; LVEDP, left ventricular end-diastolic pressure; and e, extraneous variable.

We also performed the same statistical analyses with the patients from Groups II, III, and IV. We examined the effects of LVESVI and LVEDVI on SVI (path models H-J in Fig. 4; precise data are presented in Supplemental Tables 9, 10, and 11). The results were similar to those from Group I. We also examined the effects of LVESVI, LVEDVI, and LVEDP on Log BNP in the same manner. The proposed path models K-M are illustrated in Fig. 5, and the results are presented in Supplemental Tables 12, 13, and 14, respectively. The results were similar to those from Group I, except for one path from LVEDVI to Log BNP in Group III (path model L).

### Critical ratios of differences in the significance of the increases in Log BNP between LVESVI and LVEDP

Figure 3 (path model G) and Supplemental Table 8 demonstrate that LVESVI and LVEDP were significantly associated with increases in Log BNP. Finally, we compared the powers of the effects of LVESVI and LVEDP. The critical ratios of the differences between the parameters were examined using a matrix (the full AMOS matrix is not shown because of its size). The results revealed that compared to LVEDP, LVESVI was more strongly associated with Log BNP (*P* < 0.001).

## Discussion

### How can relationships between LV cavity size and cardiac function/overload be determined?

Among the hemodynamic factors that are commonly measured during cardiac catheterization, we proposed that LVESVI and LVEDVI are especially important in terms of cardiac performance because these factors directly determine the stroke volume. In the real world, the extent of LV volume enlargement differs between the systolic and diastolic phases in patients with heart failure. The detection of possible associations between LV cavity size and both cardiac function and overload is very difficult, but it is important for improving our understanding of the basic mechanism of heart failure. Naturally, the Frank-Starling law plays a pivotal role in maintaining cardiac output in patients with heart failure, and myocardial stress or overload should theoretically be related to cardiac volume in the systolic and/or diastolic phase.

### Clear distinctions between LVESVI and LVEDVI in cardiac function/overload

In this study, we first planned to examine the effect of LVESVI and LVEDVI on SVI, that latter of which was used as a marker of cardiac function. The results of path models A and B both suggested that neither LVESVI nor LVEDVI significantly influenced SVI. Clear distinctions were drawn between the parameters in the next proposed path model (model C). It is important to note that LVESVI and LVEDVI were inversely and positively associated with SVI, respectively. We believe that these results are in absolute accordance with the Frank-Starling law and that increases in LVEDVI improve cardiac function. This study represents the first trial to successfully create an image of the Frank-Starling law on a path diagram based on covariance structure analysis.

Next, we examined the possible association between LV cavity size and cardiac overload as estimated by the plasma BNP level. Path models D and E both suggested that both LVESVI and LVEDVI augmented Log BNP. We subsequently drew the theoretical path model F and successfully demonstrated that LVESVI directly caused increases in the plasma BNP levels and that LVEDVI adaptably reduced the plasma BNP levels. The outcome for LVEDVI in path model F was the opposite of that observed in path model E. Importantly, an increase in LVEDVI decreased cardiac overload as estimated by the plasma BNP level.

In this study, the *P*-values were significant, but the R^2^ values were very low in some of the models for the determination of the plasma BNP. As previously reported, there are actually many factors that are associated with plasma BNP, including aging, gender, renal dysfunction and obesity^16, 17, 19^. It is thus logical that the R^2^ values were relatively small in this study design. Nonetheless, this analysis was planned to focus only on the effects of LVESVI and LVEDVI, and the other factors were essentially irrelevant. Moreover, this study clearly demonstrated that LVESVI is a major component in the determination of Log BNP and that LVEDVI palliated the Log BNP. Therefore, the contribution of SVI to plasma BNP logically cannot be too extensive. Indeed, understandably, the correlation of −0.137 between SVI and Log BNP was significant but relatively small (Supplemental Table 1).

**Figure 6:**
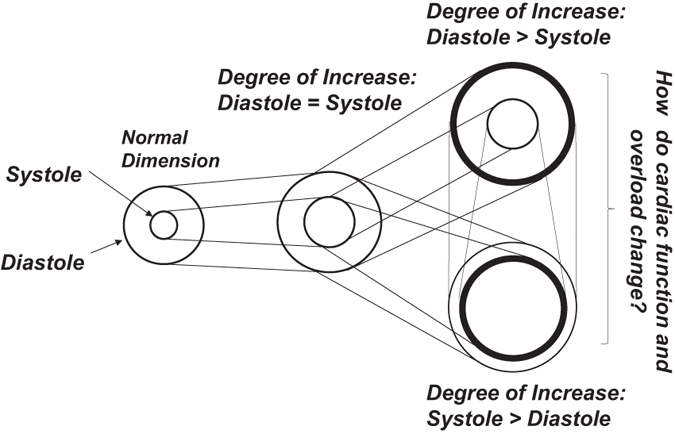
Explanatory drawing of the typical pattern of changes in the virtual cardiac shapes and the end-systolic and end-diastolic left ventricular diameters of the remodeling heart. The circles indicate the endocardium of the left ventricle in each state, the end-systolic phase and end-diastolic phase. The left ventricular cavity is dilated in the end-systolic phase and/or end-diastolic phase.

**Figure 7:**
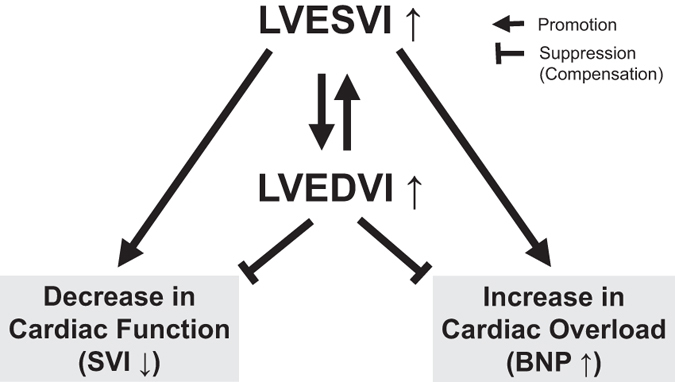
Explanatory drawing of the possible links of the hemodynamic parameters with the plasma B-type natriuretic peptide levels. The promoting and suppressing effects of these parameters on cardiac function and overload are demonstrated. BNP, B-type natriuretic peptide; LVESVI, left ventricular end-systolic volume index; LVEDVI, left ventricular end-diastolic volume index; and SVI, stroke volume index.

Figure 6 presents a graphical representation of the virtual cardiac shapes that occur during heart failure. Naturally, the patterns of enlargement in the systolic and diastolic phases differed between individuals; some patients’ LV volume increased dramatically during the end-systolic phase compared with the end-diastolic phase and vice versa. The current study clearly demonstrated that enlargement in the LV systolic phase is harmful for both cardiac function and cardiac overload, whereas enlargement in the LV diastolic phase appears to be compensatory. As illustrated in Fig. 7, when some types of heart disease occur, the cavity size increases during the systolic and diastolic phases. The different degrees of enlargement in the different phases affect cardiac function and cardiac overload in different manners.

### Analyses of the different study groups

The study population included patients with different underlying cardiac disorders. We considered it important to perform the same analyses in patients with different characteristics. We performed subgroup analyses of Groups II, III, and IV. Importantly, the results for the different groups were nearly identical, which means that the effects of LVESVI and LVEDVI on cardiac function and overload probably occur in patients with different underlying cardiac disorders. Naturally, this conjecture requires future testing with larger groups of patients with each underlying disorder.

### Clarifying the role of LVESVI as a powerful indicator of an increase in plasma BNP levels

LVESVI increased Log BNP to a greater extent than did LVEDP. This result reinforced our previous hypothesis that LVESVI is more strongly associated with the plasma BNP levels^10^.

### Directions for future studies of diastolic dysfunction

The main purpose of this study was to assess the associations between cavity size in the systolic and diastolic phases and both cardiac function and overload. We recruited the study population from the patients admitted to our hospital without selecting patients based on specific LV ejection fractions. Our results support the compensatory role of diastolic volume in cardiac function and overload. Furthermore, our results might suggest that the restriction of LV diastolic expansion is ultimately undesirable. In the future, our work will focus on clarifying the differences between the factors that are associated with heart failure according to the reduced ejection fraction and the preserved ejection fraction (HFpEF) using path models. Although the pathology and contributing factors for HFpEF have not been fully clarified, covariance structure analysis should help answer the remaining questions.

### Usable information and cautionary notes regarding the selection of the optimal surgical treatment for enlarged ventricles

LV volume reduction has become a potential surgical therapy for heart failure patients with enlarged left ventricles. Cardiac remodeling is thought to be repaired by this procedure^20, 21^. There is probably no room for doubt about the effects of volume reduction therapy in patients with significantly enlarged ventricular aneurysms owing to myocardial infarction. However, the over-indication of surgical resection for reducing systolic volume might be undesirable if the procedure results in a reduction in diastolic volume. Furthermore, such surgical methods should not be recommended without caution simply because the diastolic volume is elevated. Based on the current information, when considering surgical therapies, the portion with inadequacy in both contraction and dilatation should theoretically be the only area that is considered for surgical excision.

### Utility of covariance structure analysis in cardiovascular studies

In this study, we used covariance structure analysis to test a complicated hypothesis. This statistical method is currently used in many fields because of the widespread availability of computer processing facilities. Recently, we successfully used this method in a practical manner^19^. Path models enable us to draw conclusions about which factors we believe to underlie examined measures. This method provides more credible information than multiple regression analysis. However, the basic concept of drawing a path model is important. In this study, we successfully proposed concise and clear path models based on accumulated scientific information and our past experiences.

### Study limitations

In this study, the technique used to calculate the LV volumes from single-plane cineangiograms of the LVs is associated with significant limitations.

## Conclusion

The path model described in this report was designed to test the validity of the Frank-Starling law in patients with heart failure. When viewing plasma BNP levels as a marker of cardiac overload, LVESVI substantially augmented cardiac overload, whereas LVEDVI palliated it. The current results advance the understanding of the relationships between LV cavity size and both cardiac function and overload. The results of this study are suggestive of the increasing importance of the LV diastolic volume in patients with heart failure and the utility of the LVESVI as an important marker of cardiac remodeling for further relevant studies.

## Methods

### Study patients

The study patients consisted of 1,715 subjects who were consecutively admitted to our institution with heart disorders from 2012–2015. We simultaneously measured their plasma BNP levels and hemodynamic parameters, which included LVESVI, LVEDVI, LVEDP, and SVI, during cardiac catheterization. The LVESVI and LVEDVI were obtained from left ventriculography (LVG) traces during the end-systolic and end-diastolic phases. The contrast LVG images were acquired at a frame rate of 30 frames per second in the right anterior oblique 30-degree projection. The LVESVI and LVEDVI and the left ventricular ejection fraction were calculated from single-plane cineangiograms by means of the area-length formula using a semi-automated trace method with QAngio XA version 7.1 (Medis medical imaging systems bv, Leiden, The Netherlands). This technique for calculating LV volumes might include methodological limitations. The SVI was calculated using the thermal dilution method and a Swan-Ganz catheter. To evaluate the contributions of LV cavity size to both cardiac function and overload, we divided the patients into different groups. We performed the analyses for each group, and the groups were classified based on the following criteria: Group I consisted of all 1,715 patients who were recruited for this study, and all patients underwent left ventricular catheterization. Among these patients, right ventricular catheterization was performed in 353 patients. Group II consisted of 1,363 patients with all types of IHD. Among these patients, right ventricular catheterization was performed in 135 patients. Group III consisted of 1,080 patients with chronic stable IHD after the exclusion of those with acute coronary syndrome. Among these patients, right ventricular catheterization was performed in 133 patients. Finally, Group IV consisted of 352 patients without IHD. Among these patients, right ventricular catheterization was performed in 218 patients. The study protocol (24-355[7121]) was approved by the Ethics Committee of the Jikei University School of Medicine, and we complied with the routine ethical regulations of our institution. This was a retrospective study, and informed consent could not be obtained from each patient. Instead of obtaining informed consent from each patient, we posted a notice about the study design and contact information at a public location in our institution.

### Definition of diseases

The definitions of the diseases were as follows: In brief, IHD was diagnosed based on symptoms, electrocardiography results, blood sampling, and the morphology of the coronary arteries. The patients with IHD included those with clinically stable IHD. Organic stenosis was defined by a ≥ 75% occlusion of the coronary arteries on coronary angiography. Patients with coronary spastic angina were included in the IHD group if the disease activity was stable and a provocation test was planned during hospitalization. Valvular diseases included heart failure caused by moderate valvular disease, and patients who were scheduled for surgery were included in this group. Arrhythmia was defined by the need for catheter ablation, an implantable cardioverter-defibrillator, or cardiac resynchronization therapy, and patients with a pacemaker or syncope were included in this group. Cardiomyopathy was defined when a patient was diagnosed before admission and underwent treatment or if a patient was diagnosed after admission (excluding cases of ischemic cardiomyopathy). Infectious heart disease included pericarditis, myocarditis, and infectious endocarditis. Hypertension, diabetes mellitus, and dyslipidemia were defined as described previously^22^.

### Blood sampling and biochemical examination

We used blood sampling and hemodynamic data during the cardiac catheterization. The serum biochemical analyses and the measurements of the plasma BNP levels were performed in a central laboratory at our hospital during the study period. The plasma BNP level was measured as described in previous reports^16, 17, 19^. In brief, whole blood (5 mL) was collected in tubes containing potassium ethylenediaminetetraacetic acid (1 mg/mL blood). The plasma BNP level was then measured with a rapid enzyme-linked immunosorbent assay (non-extracted) kit using an antibody to human BNP (Shionogi Co. Ltd., Tokyo, Japan). Group III consisted of patients with chronic and stable IHD after excluding patients with acute coronary syndrome because plasma BNP levels noticeably and rapidly increase during the 24 h after the onset of acute myocardial infarction in a monophasic manner and then transiently decrease; moreover, the latter decrease is potentially followed by another increase 2–3 days after onset (depending on the degree of ventricular remodeling), which results in a biphasic profile^23^.

### Statistical analysis

Continuous variables are expressed as the means ± the standard deviations (SDs) or the medians with the ranges. Categorical variables are expressed as percentages. Comparisons between two continuous variables were performed using Pearson’s product-moment correlation coefficient analysis. The Kolmogorov-Smirnov test was used to determine whether the BNP values were normally distributed. Subsequently, the BNP data were log-transformed (Log BNP) to achieve a normal distribution for the analysis. All statistical analyses were performed using SPSS Statistics version 23.0 (SPSS Inc., Chicago, IL, USA).

A path model based on covariance structure analysis was proposed to investigate the relationships between clinical factors in the study population and specifically to identify the factors that most likely exerted causal effects on IHD. The path analyses were performed with IBM SPSS AMOS version 23 (Amos Development Corporation, Meadville, PA, USA). The obtained structural equation models were tested and confirmed at a significance level of *P* < 0.05. The causality model defined some hierarchical regressions between the hemodynamic parameters and cardiac function/overload. The paths between the variables were drawn from the independent to the dependent variables with directional arrows that were used for every regression model (i.e., arrowhead on one end only). A two-way arrow between two variables indicated a correlation between these variables. For every regression, the total variance in the dependent variable was theorized to be caused either by the independent variables in the model or by extraneous variables (e). Each path had a coefficient that indicated the standardized coefficient of the regressing independent variable on the dependent variable of the relevant path. When obtaining the critical ratios of the differences between parameters, we used AMOS to display the matrix, which included a row and a column for each parameter of the model. Each off-diagonal entry in the matrix provided a statistic for testing the hypothesis that the two-model parameters were equal in the population. The corresponding author had full access to all of the data in the study and takes responsibility for both its integrity and the data analysis.

## Electronic supplementary material

Supplementary Table 1-14
