# Supplementary material for: Associations between Left Ventricular Cavity Size and Cardiac Function and Overload Determined by Natriuretic Peptide Levels and a Covariance Structure Analysis
Source: Sci Rep. 2017 May 17;7:2037. doi: 10.1038/s41598-017-02247-5 (PMC5435711; doi:10.1038/s41598-017-02247-5)
Supplement: Supplementary file 1 — Supplementary Table 1-14 [file 41598_2017_2247_MOESM1_ESM.doc]

**Supplemental Material**

**Associations between Left Ventricular Cavity Size and Cardiac Function and Overload Determined by Natriuretic Peptide Levels and a Covariance Structure Analysis**

Jun Yoshida, M.D., Makoto Kawai, M.D., Ph.D., Kosuke Minai, M.D., Ph.D., Kazuo Ogawa, M.D., Ph.D., Takayuki Ogawa, M.D., Ph.D., Michihiro Yoshimura M.D., Ph.D.

Division of Cardiology, Department of Internal Medicine, The Jikei University School of Medicine

**Corresponding author:** Makoto Kawai, M.D., Ph.D.

Division of Cardiology, Department of Internal Medicine,

The Jikei University School of Medicine

3-25-8 Nishi-shimbashi, Minato-ku, Tokyo 105-8461, Japan.

Telephone: +81-3-3433-1111, Facsimile: +81-3-3459-6043

E-mail: cadmk@jikei.ac.jp

**Short Title:** *Yoshida* et al*. LV cavity size and cardiac function*

**Keywords**: biochemical marker, B-type natriuretic peptide, left ventricular cavity size, theoretical path model

**Supplemental Table 1**. Results of Pearson’s product-moment correlation coefficient analyses of the hemodynamic parameters

|  | LVESVI (n = 1713) | LVEDVI (n = 1713) | LVEDP (n = 1713) | SVI (n = 353) | Log BNP (n = 1713) |
| --- | --- | --- | --- | --- | --- |
| LVESVI | - | 0.898^†^ | 0.275^†^ | -0.133* | 0.501^†^ |
| LVEDVI | 0.898^†^ | - | 0.279^†^ | 0.081 | 0.414^†^ |
| LVEDP | 0.275^†^ | 0.279^†^ | - | -0.025 | 0.294^†^ |
| SVI | -0.133* | 0.081 | -0.025 | - | -0.137^†^ |
| Log BNP | 0.501^†^ | 0.414^†^ | 0.294^†^ | -0.137^†^ | - |

LVESVI, left ventricular end-systolic volume index; LVEDVI, left ventricular end-diastolic volume index; LVEDP, left ventricular end-diastolic pressure; SVI, stroke volume index; BNP, B-type natriuretic peptide. *P* value: **P* < 0.05; ^†^, *P* < 0.001; no mark, not significant.

**Supplemental Table 2.** Results of path model A (Group I): Regression coefficients and standardized regression coefficients for the entire patient group

| R^2^ = 0.009 | | | Regression coefficient | 95% CI | *P*-Value | Standardized regression coefficient | 95% CI |
| --- | --- | --- | --- | --- | --- | --- | --- |
| SVI | ← | LVESVI | -0.059 | -0.124, 0.006 | 0.072 | -0.095 | -0.168, -0.020 |

The direct effects of the theoretically proposed path model (see Figure 1).

SVI, stroke volume index; LVESVI, left ventricular end-systolic volume index; and CI, confidence interval; R^2^, squared multiple correlation.

**Supplemental Table 3.** Results from path model B (Group I)

| R^2^ = 0.004 | | | Regression coefficient | 95% CI | *P*-Value | Standardized regression coefficient | 95% CI |
| --- | --- | --- | --- | --- | --- | --- | --- |
| SVI | ← | LVEDVI | 0.031 | -0.022, 0.084 | 0.257 | 0.060 | -0.018, 0.136 |

The direct effects of the theoretically proposed path model (see Figure 1).

SVI, stroke volume index; LVEDVI, left ventricular end-diastolic volume index; and CI, confidence interval; R^2^, squared multiple correlation.

**Supplemental Table 4.** Results from path model C (Group I)

| R^2^ = 0.107 | | | Regression coefficient | 95% CI | *P*-Value | Standardized regression coefficient | 95% CI |
| --- | --- | --- | --- | --- | --- | --- | --- |
| SVI | ← | LVESVI | -0.429 | -0.562, -0.296 | <0.001 | -0.724 | -0.863, -0.569 |
|  | ← | LVEDVI | 0.350 | 0.242, 0.458 | <0.001 | 0.727 | 0.569, 0.871 |
|  |  |  | Correlation coefficient | | | | |
| LVESVI | ↔ | LVEDVI | 0.898 |  | <0.001 |  |  |

The direct effects of the theoretically proposed path model (see Figure 1).

SVI, stroke volume index; LVESVI, left ventricular end-systolic volume index; LVEDVI, left ventricular end-diastolic volume index; CI, confidence interval; R^2^, squared multiple correlation.

**Supplemental Table 5.** Results from of path model D (Group I)

| R^2^ = 0.251 | | | Regression coefficient | 95% CI | *P*-Value | Standardized regression coefficient | 95% CI |
| --- | --- | --- | --- | --- | --- | --- | --- |
| Log BNP | ← | LVESVI | 0.016 | 0.014, 0.018 | <0.001 | 0.501 | 0.465, 0.536 |

The direct effects of the theoretically proposed path model (see Figure 2).

BNP, B-type natriuretic peptide; LVESVI, left ventricular end-systolic volume index; CI, confidence interval; R^2^, squared multiple correlation.

**Supplemental Table 6.** Results from path model E (Group I)

| R^2^ = 0.171 | | | Regression coefficient | 95% CI | *P*-Value | Standardized regression coefficient | 95% CI |
| --- | --- | --- | --- | --- | --- | --- | --- |
| Log BNP | ← | LVEDVI | 0.011 | 0.009, 0.013 | <0.001 | 0.414 | 0.374, 0.453 |

The direct effects of the theoretically proposed path model (see Figure 2).

BNP, B-type natriuretic peptide; LVEDVI, left ventricular end-diastolic volume index; CI, confidence interval; R^2^, squared multiple correlation.

**Supplemental Table 7.** Results from path model F (Group I)

| R^2^ = 0.258 | | | Regression coefficient | 95% CI | *P*-Value | Standardized regression coefficient | 95% CI |
| --- | --- | --- | --- | --- | --- | --- | --- |
| Log BNP | ← | LVESVI | 0.022 | 0.018, 0.026 | <0.001 | 0.669 | 0.580, 0.756 |
|  | ← | LVEDVI | -0.005 | -0.007, -0.003 | <0.001 | -0.187 | -0.278, -0.094 |
|  |  |  | Correlation coefficient | | | | |
| LVESVI | ↔ | LVEDVI | 0.898 |  | <0.001 |  |  |

The direct effects of the theoretically proposed path model (see Figure 2).

BNP, B-type natriuretic peptide; LVESVI, left ventricular end-systolic volume index; LVEDVI, left ventricular end-diastolic volume index; CI, confidence interval; R^2^, squared multiple correlation.

**Supplemental Table 8.** Results from path model G (Group I)

| R^2^ = 0.286 | | | Regression coefficient | 95% CI | *P*-Value | Standardized regression coefficient | 95% CI |
| --- | --- | --- | --- | --- | --- | --- | --- |
| Log BNP | ← | LVESVI | 0.021 | 0.017, 0.025 | <0.001 | 0.647 | 0.558, 0.733 |
|  | ← | LVEDVI | -0.006 | -0.008, -0.004 | <0.001 | -0.215 | -0.306, -0.124 |
|  | ← | LVEDP | 0.013 | 0.009, 0.017 | <0.001 | 0.176 | 0.134, 0.217 |
|  |  |  | Correlation coefficient | | | | |
| LVESVI | ↔ | LVEDVI | 0.898 |  | <0.001 |  |  |
| LVESVI | ↔ | LVEDP | 0.275 |  | <0.001 |  |  |
| LVEDVI | ↔ | LVEDP | 0.279 |  | <0.001 |  |  |

The direct effects of the theoretically proposed path model (see Figure 3).

BNP, B-type natriuretic peptide; LVESVI, left ventricular end-systolic volume index; LVEDVI, left ventricular end-diastolic volume index; LVEDP, left ventricular end-diastolic pressure; CI, confidence interval. R^2^: squared multiple correlations.

**Supplemental Table 9.** Results from path model H (Group II)

| R^2^ = 0.147 | | | Regression coefficient | 95% CI | *P*-Value | Standardized regression coefficient | 95% CI |
| --- | --- | --- | --- | --- | --- | --- | --- |
| SVI | ← | LVESVI | -0.568 | -0.815, -0.321 | <0.001 | -0.776 | -0.985, -0.511 |
|  | ← | LVEDVI | 0.489 | 0.289, 0.689 | <0.001 | 0.821 | 0.531, 1.049 |
|  |  |  | Correlation coefficient | | | | |
| LVESVI | ↔ | LVEDVI | 0.886 |  | <0.001 |  |  |

The direct effects of the theoretically proposed path model (see Figure 4).

SVI, stroke volume index; LVESVI, left ventricular end-systolic volume index; LVEDVI, left ventricular end-diastolic volume index; CI, confidence interval; R^2^, squared multiple correlation.

**Supplemental Table 10.** Results of path model I (Group III)

| R^2^ = 0.145 | | | Regression coefficient | 95% CI | *P*-Value | Standardized regression coefficient | 95% CI |
| --- | --- | --- | --- | --- | --- | --- | --- |
| SVI | ← | LVESVI | -0.574 | -0.823, -0.325 | <0.001 | -0.803 | -1.015, -0.531 |
|  | ← | LVEDVI | 0.488 | 0.284, 0.692 | <0.001 | 0.834 | 0.537, 1.068 |
|  |  |  | Correlation coefficient | | | | |
| LVESVI | ↔ | LVEDVI | 0.893 |  | <0.001 |  |  |

The direct effects of the theoretically proposed path model (see Figure 4).

SVI, stroke volume index; LVESVI, left ventricular end-systolic volume index; LVEDVI, left ventricular end-diastolic volume index; CI, confidence interval; R^2^, squared multiple correlation.

**Supplemental Table 11.** Results from path model J (Group IV)

| R^2^ = 0.178 | | | Regression coefficient | 95% CI | *P*-Value | Standardized regression coefficient | 95% CI |
| --- | --- | --- | --- | --- | --- | --- | --- |
| SVI | ← | LVESVI | -0.403 | -0.521, -0.285 | <0.001 | -0.914 | -1.125, -0.676 |
|  | ← | LVEDVI | 0.331 | 0.233, 0.429 | <0.001 | 0.912 | 0.673, 1.124 |
|  |  |  | Correlation coefficient | | | | |
| LVESVI | ↔ | LVEDVI | 0.894 |  | <0.001 |  |  |

The direct effects of the theoretically proposed path model (see Figure 4).

SVI, stroke volume index; LVESVI, left ventricular end-systolic volume index; LVEDVI, left ventricular end-diastolic volume index; CI, confidence interval; R^2^, squared multiple correlation.

**Supplemental Table 12.** Results from path model K (Group II)

| R^2^ = 0.286 | | | Regression coefficient | 95% CI | *P*-Value | Standardized regression coefficient | 95% CI |
| --- | --- | --- | --- | --- | --- | --- | --- |
| Log BNP | ← | LVESVI | 0.026 | 0.022, 0.030 | <0.001 | 0.654 | 0.559, 0.746 |
|  | ← | LVEDVI | -0.007 | -0.011, -0.003 | <0.001 | -0.224 | -0.321, -0.126 |
|  | ← | LVEDP | 0.012 | 0.008, 0.016 | <0.001 | 0.161 | 0.114, 0.209 |
|  |  |  | Correlation coefficient | | | | |
| LVESVI | ↔ | LVEDVI | 0.887 |  | <0.001 |  |  |
| LVESVI | ↔ | LVEDP | 0.298 |  | <0.001 |  |  |
| LVEDVI | ↔ | LVEDP | 0.288 |  | <0.001 |  |  |

The direct effects of the theoretically proposed path model (see Figure 5).

BNP, B-type natriuretic peptide; LVESVI, left ventricular end-systolic volume index; LVEDVI, left ventricular end-diastolic volume index; LVEDP, left ventricular end-diastolic pressure; CI, confidence interval; R^2^, squared multiple correlation.

**Supplemental Table 13.** Results of path model L (Group III)

| R^2^ = 0.296 | | | Regression coefficient | 95% CI | *P*-Value | Standardized regression coefficient | 95% CI |
| --- | --- | --- | --- | --- | --- | --- | --- |
| Log BNP | ← | LVESVI | 0.021 | 0.017, 0.025 | <0.001 | 0.559 | 0.450, 0.666 |
|  | ← | LVEDVI | -0.003 | -0.007, 0.001 | 0.067 | -0.104 | -0.213, 0.008 |
|  | ← | LVEDP | 0.012 | 0.008, 0.016 | <0.001 | 0.172 | 0.119, 0.224 |
|  |  |  | Correlation coefficient | | | | |
| LVESVI | ↔ | LVEDVI | 0.893 |  | <0.001 |  |  |
| LVESVI | ↔ | LVEDP | 0.296 |  | <0.001 |  |  |
| LVEDVI | ↔ | LVEDP | 0.292 |  | <0.001 |  |  |

The direct effects of the theoretically proposed path model (see Figure 5).

BNP, B-type natriuretic peptide; LVESVI, left ventricular end-systolic volume index; LVEDVI, left ventricular end-diastolic volume index; LVEDP, left ventricular end-diastolic pressure; CI, confidence interval; R^2^, squared multiple correlation.

**Supplemental Table 14.** Results from path model M (Group IV)

| R^2^ = 0.256 | | | Regression coefficient | 95% CI | *P*-Value | Standardized regression coefficient | 95% CI |
| --- | --- | --- | --- | --- | --- | --- | --- |
| Log BNP | ← | LVESVI | 0.015 | 0.011, 0.019 | <0.001 | 0.649 | 0.449, 0.839 |
|  | ← | LVEDVI | -0.005 | -0.009, -0.001 | 0.008 | -0.274 | -0.477, -0.066 |
|  | ← | LVEDP | 0.017 | 0.009, 0.025 | <0.001 | 0.205 | 0.108, 0.298 |
|  |  |  | Correlation coefficient | | | | |
| LVESVI | ↔ | LVEDVI | 0.894 |  | <0.001 |  |  |
| LVESVI | ↔ | LVEDP | 0.257 |  | <0.001 |  |  |
| LVEDVI | ↔ | LVEDP | 0.296 |  | <0.001 |  |  |

The direct effects of the theoretically proposed path model (see Figure 5).

BNP, B-type natriuretic peptide; LVESVI, left ventricular end-systolic volume index; LVEDVI, left ventricular end-diastolic volume index; LVEDP, left ventricular end-diastolic pressure; CI, confidence interval;. R^2^, squared multiple correlation.
